# 3D Electron Diffraction for Chemical Analysis: Instrumentation Developments and Innovative Applications

**DOI:** 10.1021/acs.chemrev.1c00207

**Published:** 2021-09-17

**Authors:** Tim Gruene, Enrico Mugnaioli

**Affiliations:** † University of Vienna, Faculty of Chemistry, Department of Inorganic Chemistry, AT-1090 Vienna, Austria; ‡ Center for Nanotechnology Innovation@NEST, Istituto Italiano di Tecnologia, Piazza S. Silvestro 12, IT-56127 Pisa, Italy

## Abstract

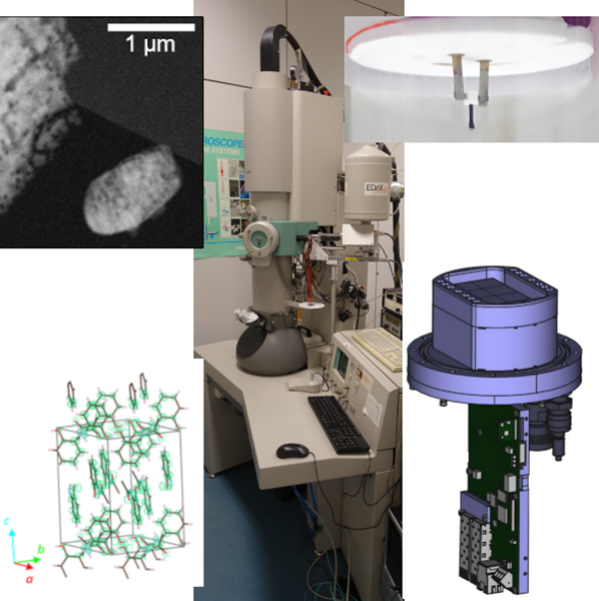

In the past few years, many exciting papers reported results based on crystal structure determination by electron diffraction. The aim of this review is to provide general and practical information to structural chemists interested in stepping into this emerging field. We discuss technical characteristics of electron microscopes for research units that would like to acquire their own instrumentation, as well as those practical aspects that appear different between X-ray and electron crystallography. We also include a discussion about applications where electron crystallography provides information that is different, and possibly complementary, with respect to what is available from X-ray crystallography.

## 1 Introduction

Electron crystallography is gaining acceptance as a method of structure elucidation in chemistry, structural chemistry, and materials science, complementary to X-ray crystallography. In 2018, two publications about structure determination of organic compounds with electron diffraction^1,2^ triggered a lot of attention. Matthew Warren asked “Why didn’t we think to do this earlier? [...]”^3^ With a better account for the developments in 3D electron diffraction (3D ED), one would better phrase this as “Why didn’t we realize this earlier?”, because chemical structure determination with electron diffraction has a much longer history. The first paper^1^ is in fact the main publication of the research project “Applicability of 3D electron diffraction in the pharmaceutical industry” (A3EDPI). The motivation for this project proposal was the realization that the majority of crystallographers, and chemists who make use of crystallography, were even unaware of the existence of the 3D ED method. The project proposal aimed at changing this lack of awareness.

Several recent reviews light the field from various angles. Nannenga^4^ and Clabbers and Xu^5^ cover the sample preparation, data processing, and refinement process, with an emphasis on organic and macromolecular compounds. Gemmi et al.^6^ have provided an overview of the historical development, the various kinds of data collection techniques, and examples from a broad range of applications. Huang et al.^7^ describe a long history of electron diffraction in the field of metal organic and covalent organic frameworks (MOFs and COFs).

The name “3D ED” was suggested by Gemmi et al.^6^ as an umbrella term for a number of techniques that collect reflection intensities in the 3-dimensional reciprocal space by an electron diffraction experiment. Several very similar techniques have been developed in the past 15 years. The very original term is automated diffraction tomography (ADT^8^); the most popular term is probably microcrystal electron diffraction (microED^9^). Other terms include rotation electron diffraction (RED^10^), continuous rotation electron diffraction (cRED^11^), electron diffraction tomography (EDT^12^), integrated electron diffraction tomography (IEDT^13^), and precession electron diffraction tomography (PEDT^14^).

The crystal size is the most striking difference between electron and X-ray diffraction, especially for X-ray crystallographers who are new to ED. Due to the strong interaction between electrons and matter, crystals can be as small as a few tens of nanometers and up to about 1 μm thick. Between 1 and 5 μm, depending on the elemental composition of the crystals, electrons with an energy of 200 keV get absorbed, so that ED closes the gap to X-ray crystallography, where 5 μm is about the lower end of what can be measured with single crystal X-ray diffraction. This enables the analysis of minute single crystals that compose powder samples. Individual single crystal structures can be determined from powder blends. This resolves the ambiguity that X-ray powder diffraction can suffer from. The importance of this was recently recognized in the extensive study of 50 organic samples by Bruhn et al.^15^ and readily realized during the study of a new lithium-rich zincolithosilicate.^16^ The strong interaction of electrons with matter leads to a significant deviation of the Bragg intensities from the kinematic theory of diffraction. Ignoring this effect, i.e. applying a kinematic refinement, yields already fairly good results.^15^ More details can be revealed with dynamical refinement that takes the dynamical theory of diffraction into account. Although this slows down the refinement process, dynamical refinement makes ED very sensitive for the determination of chirality and the location of hydrogen.^17,18^ We refer the reader to refs (19−21) for a detailed discussion and to ref (22) for an overview of dynamical refinement and its possibilities in ED.

The present review focuses on instrumental aspects for chemists who wish to include ED as an analytical tool in crystallography. It complements the perspective of Gruene et al.,^22^ which addresses the experimental aspects of sample preparation and the data analysis process, and it complements the review of Gemmi et al.,^6^ which covers the broad field of applications of ED, explains the different types of data collection strategies, and summarizes the historical development of 3D ED. This paper also adds some of the most recent results since Gemmi et al.^6^

## 2 Access to Electron Diffraction

Any lab that carries out X-ray diffraction has, in principle, the expertise to carry out electron diffraction too. The costs for an instrument suitable for electron diffraction are of the same order of magnitude as a modern X-ray diffractometer. The skill required to operate a transmission electron microscope for electron diffraction is comparable to the skill required to operate an X-ray diffractometer. The same software for all steps of data analysis can be used, although programs specifically dedicated to electron diffraction are available.^23^ The main difference is the possibility of taking dynamical scattering into account during structure refinement. Dynamic refinement results in a higher level of detail for the interpretation of the structure, e.g. a better observation of hydrogen atoms.^17^ It can also be used to determine the chirality of chiral molecules.^18^

Table 1 is a compilation of laboratories where electron diffraction is carried out. It has been compiled from the structures deposited at the Cambridge Crystallographic Database, CCDC, by searching for the keyword “electron diffraction”,^24,25^ and from recent publications. Table 1 is likely bound to be incomplete. The fact that the number of laboratories still fits into a table illustrates that 3D ED has not unfolded its potential, yet.

**Table 1:** Collection of Institutes That Recently Carried out and Published Structures Determined from 3D Electron Diffraction

**Institute**	**PI**	**refs**
Czech Academy of Sciences (CZ)	L. Palatinus	
	P. Brazda	
	G. Steciuk	(26, 27)
Univ. of California, Los Angeles (US)	T. Gonen	(2, 28)
	H. Nelson	(29, 30)
	J. A. Rodriguez	(2, 31)
Univ. of Stockholm (SE)	X. Zou	(32, 33)
	H. Xu	(5, 34)
Univ. of Vienna (AT)	T. Gruene	(1, 35)
Nanoimaging Services (US)	J. Bruhn	(15, 36)
Univ. of Tokyo (JP)	E. Nakamura	(37)
Paul Scherrer Inst. (CH)	E. Müller Gubler	(1)
	E. Poghosyan	
	J. P. Abrahams	(38, 39)
RIKEN SPring-8 Centre (JP)	K. Yonekura	(40, 41)
	S. Maki-Yonekura	
Univ. of Hamburg (DE)	R. Bücker	(42)
Center for Nanotechnology Innovation, Pisa (IT)	M. Gemmi	(43, 44)
	E. Mugnaioli	
Electron Bio-Imaging Centre (eBIC) (UK)	P. Zhang	(45, 46)
	D. G. Waterman	(15)
Johannes Gutenberg Univ. Mainz (DE)	U. Kolb	(47, 48)
CNRS ENSICAEN (FR)	P. Boullay	(17, 49)
Univ. of Antwerpen (BE)	J. Hadermann	(50)
Univ. Grenoble Alpes and CNRS (FR)	S. Kodjikian	(51, 52)
	H. Klein	
Univ. of Manchester (UK)	A. S. Eggemann	(53)
Arizona State Univ. (US)	B. Nannenga	(54)
Univ. Lille (FR)	D. Jacob	(21, 55)

## 3 Instrumentation

X-ray crystal structures are determined with X-ray diffractometers. Electron diffractometers, in an analogue sense, are currently under development. Vainshtein presented their horizontal electron diffraction chamber in his book on electron diffraction in 1964,^56^ but it appears that a commercial solution has never existed. There are now two commercial electron diffractometers under development. Both ELDICO-Scientific and Rigaku have presented a first model of their instruments. The control software of both instruments is close to what X-ray crystallographers are familiar with from their X-ray home sources.^57,58^ Rigaku presented their electron diffractometer XtaLAB Synergy-ED in spring 2021. It is based on a JEOL JEM2100Plus and fully controlled through the Rigaku CrysAlis Pro software, which also operates their X-ray diffractometers.^58^ The instrument by ELDICO is an entirely new design, much more akin to an X-ray diffractometer. The Swiss Innovation Park Basel Area also expects a dedicated electron diffractometer during summer 2021 (priv. commun. Dr. G. Santiso-Quĩnones, ELDICO Scientific).

Up to now, transmission electron microscopes (TEMs) have been used instead of electron diffractometers. Two manufacturers of TEMs, Thermofisher and JEOL, offer “microED” packages as software upgrades for their instruments. This mainly addresses the need in crystallography to rotate the crystal about an axis with a constant angular velocity. Hitachi is another TEM manufacturer, although we are not certain whether extensions for 3D electron diffraction are available. It should be noted that several freely available software solutions such as Instamatic/Insteadmatic,^59,60^ parallelEM,^41,61^ fast-ADT,^62^ and a collection of scripts based on serialEM^63^ are independent of such “microED” packages. SerialEM is a generic software interface, which works on a broad range of instruments.^64^

Unlike X-ray diffractometers, TEMs usually have a single axis goniometer. This axis is typically called the α-axis. Depending on the make, the sample-holder can have a second, β-axis for positioning the sample at an angle nonperpendicular to the incident beam. However, in our experience, the β-axis is not eucentric, and crystal alignment becomes rather difficult.

### 3.1 Choice of Electron Source: *LaB*_6_ or FEG

TEMs can be equipped with two types of electron source: thermo-ionic cathodes, such as a Tungsten (*W*) hairpin or a pointed lanthanum hexabromide (*LaB*_6_) crystal, or field emission guns (FEGs).^65^ TEMs with a thermo-ionic electron source are more commonly found and can readily be used for 3D ED, as long as the rotation range of the goniometer is reasonably wide.

The energy bandwidth is about 0.2–0.7 eV for a FEG and 1–2 eV for a *LaB*_6_ source. At 200 keV, the energy spread  of the *LaB*_6_ source corresponds to the energy spread of a perfect silicon monochromator used at X-ray synchrotron beamlines.^66^ Thus, for electron diffraction the advantages of a FEG source are not as striking as for imaging, and a TEM equipped with an *LaB*_6_ source can deliver comparable results at a significantly smaller cost and easier maintenance. A modern, high-end TEM with an *LaB*_6_ source with energy varying from 80 to 200 keV is available for less than 800 k€. This is of the same order of magnitude as a modern X-ray diffractometer (400–500 k€). Refurbished second-hand, good quality TEMs are available for about 200–300 k€. Moreover, an *LaB*_6_ electron source is much less sensitive to damage from impacting positive ions and requires a vacuum *P* ≤ 10^–6^ mbar. FEGs require at least *P* ≤ 10^–8^–10^–10^ mbar.^65^

The divergence of the electron beam of a *LaB*_6_ source is about the same order of magnitude as the X-ray beam of a third-generation synchrotron and at least 1 order of magnitude less than the X-ray beam of an in-house X-ray diffractometer. This is sufficient for most crystallographic applications, and the lower divergence (“greater parallelity”) of FEGs brings no extra benefit.

On the other hand, the coherence length for FEGs is much larger than for *LaB*_6_. The electron beam for a FEG allows probing the sample with a quasi-parallel beam of a few tens of nanometers, which results advantageous for the study of nanoparticles, multiphase nanodevices, precipitates casted on crystalline matrices, and materials characterized by nanotwinning or aggregated nanodomains. Moreover, the higher brilliance of an FEG can improve the performance of serial and roto-serial diffraction acquisitions (see section 4.4).

Independent of the electron source, all TEMs can be equipped with an energy filter. Energy filters significantly reduce the background scatter,^67^ especially at low voltage. However, background subtraction is well advanced in data reduction programs,^68^ so that an energy filter produces visually appealing diffraction patterns, but the experimental proof of better data quality is not available to date. A recent theoretical study suggests that an energy filter may actually enhance the relative contribution of dynamical scattering.^69^ However, hitherto no experimental data in support of this effect have been produced.

In addition to the above technical aspects, high-end TEMs are available with an “autoloader”, a cryogenic sample manipulation robot, which may be convenient for high-throughput screening of cryo-protected samples. In our experience, though, the time-limiting step is crystal search rather than changing grids, and we would not consider an autoloader a “must-have” accessory.

### 3.2 Choice of Energy, 120 keV vs 200 keV vs 300 keV

There are instruments available with a maximum acceleration voltage of 120 kV, 200 kV, or 300 kV. The choice is mainly a matter of costs and of accessories, that may be available for a high-end TEM at 300 kV equipped with a FEG but not a midrange 120 kV or 200 kV TEM with a *LaB*_6_ electron source. In addition to financial considerations, the penetration depth of 300 keV electrons is slightly greater than at 200 keV so that thicker samples can be studied. The difference in cross section, however, is rather small and can be compensated with preparing smaller crystals.^70,71^ The upper limit for organic crystals is about 1 μm at 200 keV. As the thickness increases, the diffraction pattern becomes fuzzy, before the electrons are fully absorbed from a few micrometer thick crystal. Like with X-rays, absorption increases with heavier weight elements but remains within the same order of magnitude.

The wavelength of the diffraction energy is related to the acceleration voltage of the electron beam: the energy of the electron beam equals the elementary charge (1 *e*) times the voltage. The wavelength λ results from the de Broglie equation (where *c* = 299, 792 km/s is the speed of light, *h* = 6.626070 × 10^–34^ J s is the Planck constant, *m*_0_ = 510.999 keV/*c*^2^ is the rest mass of the electron, *E* is the total energy *E*_*V*_ + *m*_0_*c*^2^, and *E*_*V*_ is the potential energy, i.e. e*voltage, measured in keV).
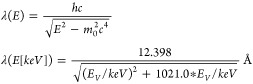


The common energies 120, 200, and 300 keV correspond to the wavelengths 0.03349 Å, 0.02508 Å, and 0.01969 Å, respectively. Note that the 2θ ranges for a typical data set with a resolution range between 0.8 and 25 Å for these three wavelengths are 2.4°–0.08°, 1.8°–0.06°, and 1.4°–0.05°, respectively. For Mo Kα X-rays, λ = 0.7107 Å, the range is 52.7°–1.6°. This requires a much larger detector distance. This distance is set only virtually by the electromagnetic lens system of the TEM. Higher energies result in a higher penetration width and reduced dynamical scattering.^40^ The effect, however, is not dramatic, and there have been excellent results at 120 keV.^17,43,44^ A 300 kV TEM can also be operated at any lower energy and thus covers a 120 kV and 200 kV TEM, likewise for a 200 keV TEM. The different setting requires a realignment of the optics and is rarely done. Lower energies result in less spread of the electrons in the detector surface and better focused intensity spots, and indeed, hybrid pixel detectors are nearly ideal at about 120 keV.^72−74^

The energy of the electron beam also slightly affects the radiation damage during sample search and data collection. The effect is only small and sample dependent and does not provide a clear-cut case for an optimal energy. At energies below 80 keV, so-called knock-on effects cannot take place for carbon. The limit is 240 keV for silicon. Knock-on refers to the kicking out of the entire atom. However, even at higher energies, knock-on effects only play a small role for radiation damage. The major contribution to radiation damage comes from radiolysis, even if the specific radiolysis damage (deposited energy per elastic scattering) is orders of magnitude lower for electrons than for X-rays.^75^ The impacting electrons trigger the release of secondary electrons from the compound, which are the main cause of damage. The physics of radiation damage by both knock-on and radiolysis are detailed in various textbooks.^65,76,77^ Radiolysis by electrons is similar to X-rays and can be reduced by cooling the sample to cryogenic temperatures.^78−80^ With X-ray diffraction, radiation damage is a major concern for macromolecular compounds and supramolecular compounds but much less so for the typical organic small molecule sample and certainly not for inorganic compounds. The minute crystal volume makes radiation damage a concern for a much larger class of materials in electron diffraction—many electron microscopists consider zeolites as radiation sensitive. A good training in macromolecular crystallography helps to appreciate a fast working mode and helps to understand the fact that looking at the crystal means damaging the crystal. Hence, wherever possible, instrument alignment is carried out with the crystal just off the field of view and only moved in position at the beginning of data collection.

### 3.3 Detectors for ED

ED patterns are traditionally recorded by charge-coupled device (CCD) cameras. Due to the limited dynamical range and the sensitivity, a beam stop is normally required not to damage such detectors after intensive ED data collections. The read-out time and the read-out noise of CCD cameras may also hinder full data acquisition from very beam-sensitive materials (like pharmaceuticals or macromolecules) before significant beam damage is introduced. Complementary metal–oxide–semiconductor (CMOS) detectors have the significant advantages of better sensitivity, lower background, and faster read-out compared to CCD cameras. Most CMOS detectors are not specifically designed for ED experiments and may suffer a certain deterioration after extensive diffraction data experiments. Still, many CMOS detectors are available with full integration into the instrument software control, and many groups collect their ED data with CMOS detectors. Companies that offer such CMOS detectors include TVIPS, Thermofisher, Gatan, and Direct Electron.^40,54,81−85^

On the other hand, hybrid pixel detectors (HPDs) have been dominating the field of X-ray crystallography since their introduction about two decades ago.^86^ Nowadays, they are abundant at synchrotron beamlines for crystallography, both for powder and for single crystal diffraction, and they also become more and more popular with in-house X-ray diffractometers. Their excellent suitability for the detection of electrons was investigated soon after.^72^ Both with X-rays and with electrons, HPDs stand out with their high dynamic range of typically 20 bit or more, zero read-out time, zero read-out noise, and high image rate above 1 kHz. In addition, with electron radiation, HPDs are radiation hard and do not require a beamstop: even long time exposure to the direct beam with electrons at 200 keV and below creates no damage, and they even stand at 300 keV, when the exposure time and beam intensity are not extreme.^73^ The absence of a beam stop facilitates data processing, as the direct beam position can be read directly from the diffraction images. HPDs are excellent also for imaging, and crystals are visible at very low beam intensity, comparable to STEM imaging,^65^ with the advantage of a live, jitter-free view during sample search.^87^ Several companies offer hybrid pixel detectors for TEMs: ASI, DECTRIS, Quantum Detectors, Rigaku, and X-Spectrum.^88−92^ In addition to these commercially available products, the JUNGFRAU detector, developed at the PSI Switzerland, was recently used to discriminate silicon from aluminum in ED data from aluminosilicates. The JUNGFRAU detector is a charge integrating detector designed for studies at free electron lasers. It can be operated with a 1 kHz or 2 kHz frame rate. Each pixel switches its gain automatically when a certain charge threshold is reached. This way it covers a very large dynamic range of 120 MeV per pixel and frame. For data processing, 50 or 100 frames, say, can be summed to get an effective frame rate of 100 Hz. This still yields very fine-sliced frames and greatly reduces the data volume.^35^ Readers interested in a JUNGFRAU detector can contact the PSD Detector group at PSI (https://www.psi.ch/en/detectors).

In terms of data quality, all of the mentioned hybrid pixel detectors are properly calibrated and have a proper gain setting. Improper gain correction leads to pixels with negative pixel counts. While the use of negative Bragg intensities from profile fitting do contain valuable information for the diffraction experiment,^93^ raw pixels with negative intensities do not make sense. They require a work-around solution or scripts to scan for the proper pedestal setting.^15,81,94,95^

The total number of pixels is usually smaller for hybrid pixel detectors than for imaging detectors, e.g. 512 × 512 pixels for the Dectris QUADRO or ASI Timepix2 versus 4k × 4k for a Gatan OneView or the Thermofisher Ceta-D detector. However, 512 × 512 pixels is sufficient for high-resolution small molecule diffraction. For protein diffraction studies, a low- and high-resolution scan can be combined, as should be good practice anyway.^96^ In our pilot study with the JUNGFRAU detector,^35^ the detector area was limited to 340 pixels across due to an aperture of a film box in the aged TEM, yet it collected complete data across the entire resolution range of 12.3–0.68 Å of zeolite A.^35^

## 4 Setting up for Data Collection

The principles of data collection are rather similar between X-ray diffraction and 3D ED. Two significant differences are the fact that the sample chamber of the TEM is under vacuum and that centering of the crystal is carried out at a much smaller scale. Both aspects are covered in the following. An additional aspect is the difference between nanobeam electron diffraction (NBD, also called nanoelectron diffraction, NED) and selected area electron diffraction (SAED). These two modes, their advantages, and disadvantages are explained very comprehensibly in Lanza et al.,^43^ especially section 3.1.

### 4.1 Working under Vacuum

Electrons are absorbed by air. The flight tube of a TEM is under vacuum. Usually, the pressure is below 10^–6^ mbar. Many types of crystals will deteriorate under these conditions and need to be protected. Crystals with solvent in their channels are particularly sensitive to vacuum. Solvent is always present in macromolecular compounds and is very common in supramolecular compounds. Organic and inorganic porous materials can also host guest molecules in their channels, which may be critical for the stability of the framework structure or may be the very topic of interest. In the early stages of cryo-EM development, molecules were typically coated by sugar, while more recently cryo-plunging emerged as the technique of choice for the study of vacuum-sensitive materials.^97^ Both techniques immobilize the sample and preserve it from vacuum deterioration. In principle, one can also use ionic liquids with very low vapor pressure, which do not evaporate under high vacuum. Ionic liquids are interesting, because they are conductive and may have a positive effect with respect to radiation damage. They also enhance the visibility of the crystals, because their electro-optical density differs more strongly from organic compounds than aqueous solutions differ from organic compounds. Another option are liquid-cell holders, where the crystals can be measured under local ambient conditions up to 1 bar.^50^ A recent review goes into greater depth about these different possibilities of sample preparation.^98^ Finally, the crystals can be immersed into a solidifying material and the crystal can be cut out with a focused ion beam (FIB milling).^45^ FIB milling can even improve the diffraction quality of the crystal. In any case, one needs to ensure that the crystals are embedded in a film thin enough to avoid absorption of the electron beam. Sample preparation is often a trial-and-error approach, and the success-rate improves with experience.^5^

### 4.2 Centering of the Crystal

In X-ray diffraction, the crystal must be centered at the intersection point of the rotation axes of the goniometer. Due to imperfections, the intersection point is actually a little volume. The X-ray beam has to be aligned to also cross this point of intersection. In electron diffraction, there is (currently) only one rotation axis, and the beam can, in principle, be shifted in position and focused at different heights. As a terminology, the word “eucentric” height is used in microscopy (eu-: representing Greek *ευ*-, combining form of *ευζ* good, used in neuter form *ευ* as adverb = well). However, when working with a submicrometer sized beam and a submicrometer sized crystal, the requirements to the stability of the goniometer are much higher than for a goniometer used in X-ray diffractometers. Furthermore, exposure of the crystal to the beam damages it, and centering should be done with the crystal translated along the rotation axis just outside of the beam. In general, the goniometer can be balanced to one particular sample holder. Ideally, a crystal can be aligned such that it stays even within a very small beam for the entire rotation range supported by the goniometer. In most instruments, this range is ≈ ±70° from the horizontal of the grid plane.

**Figure 1:**
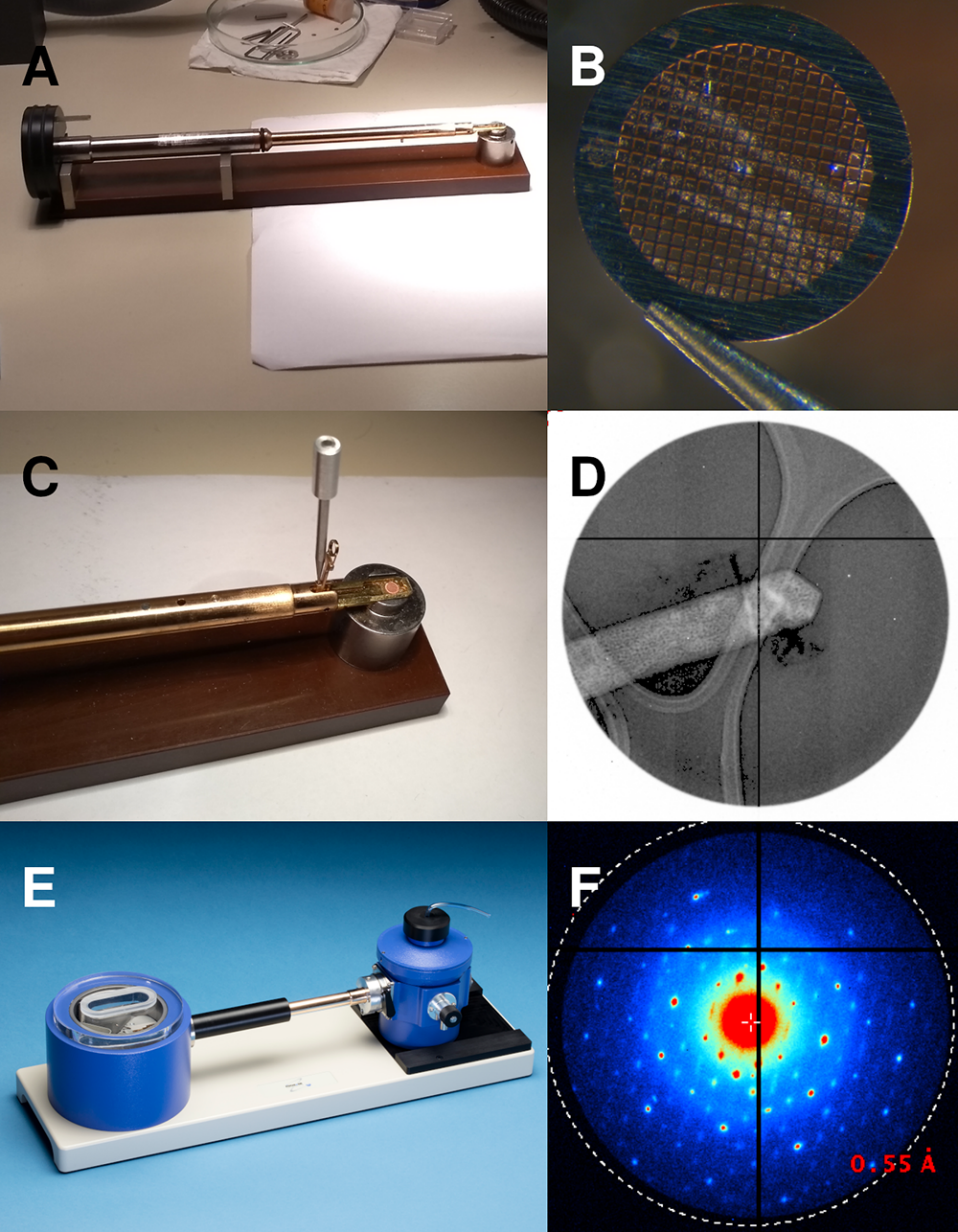
Holder must be balanced to the goniometer for stable rotation. (A) Room-temperature holder used in Vienna. (B) Typical TEM sample grid, 3.05 diameter, covered with crystalline powder. (C) Opened clip of the holder with grid already placed. (D) Sample crystal with a beam diameter of 750 nm. The crystal is 125 nm wide; the visible length is 500 nm (courtesy J. T. C. Wennmacher). (E) Example of a cryo-transfer holder inside the cryo-transfer station (Fischione Model 2550, with kind permission). (F) The well-aligned crystal (D) diffracts throughout 80° rotation (the maximum possible with the CM200 in Vienna; courtesy J. T. C. Wennmacher).

Figure 1 shows a room-temperature holder and a cryo-tomography holder. The room-temperature holder is balanced with the goniometer at the TEM in Vienna. A 500 nm crystal stays inside a 750 nm beam diameter throughout 80°, the maximum rotation range of this instrument. Using a different holder disturbs the balance. This can reduce the total available rotation range. The same needs to be taken into consideration for cryo-holders when the liquid nitrogen evaporates with time. One solution to unstable goniometers is to track the crystal. This can be done before data collection with a “dummy rotation” in imaging mode,^62^ or during data collection, by switching the instrument to imaging mode in regular intervals and discarding the respective frames from data processing.^59^ However, switching between imaging and diffraction mode may suffer from hysteresis effects of the electromagnetic lenses and is therefore not suitable for all instruments. A third option, which was actually developed long before continuous rotation data collection was introduced to ED by Nannenga et al.^9^ and consists of the combination of still images with beam precession at each position,^99−101^ cf. section 4.3.

Once the sample grid has been centered near the crystal, the instrument can be set from imaging mode to diffraction mode. Electron microscopes feature a simple push button for this. With a perfect instrument, the diffraction pattern is focused on the detector plane, resulting in optimally focused Bragg spots. When the direct beam is visible, as is possible with HPDs, the pattern can be focused manually with the focus button. This process takes only a few seconds. For detectors that get damaged from exposure to the strong direct beam, this can be done at low beam intensity, before placing the beam stop. Once the crystal is centered and the beam is focused, data collection is started as with X-ray diffraction; that is, the crystal is set to rotate, and the detector starts to collect data.

### 4.3 Data Collection with Precession vs Rotation

Data collection with the rotation method consists of the rotation of the crystal on a single axis. Ideally, the crystal volume is evenly illuminated throughout the rotation. The rotation method as described by Arndt and Wonacott^102^ results in a *contiguous* section of reciprocal space. Later, when detectors became available with negligible readout-time, contiguous data collection turned into continuous, i.e. shutterless data collection.^103^

3D ED data collection uses a beam diameter of usually less than 1.5 μm. Some instruments permit a beam diameter down to 30 nm. This is instrument specific and affected by the diameter of the condenser lens aperture. In electron diffraction, the rotation range is usually limited to a maximum of about 150°. At angles greater than ±75° off the horizontal, the sample holder blocks the beam. This rotation range sets high requirements to the mechanical reliability of the goniometer. The crystal must be centered to within half the beam diameter to ensure it does not leave the beam during exposure. When the crystal is very small, and therefore the beam diameter is very small, or when the sample holder is not very well balanced with respect to the instrument goniometer, the crystal is likely to move out of the beam during rotation. When the holder itself is not calibrated well to the goniometer, it may not be possible at all to correct for the drift out of the beam. As a compromise, the beam diameter can be increased, at the cost of increased background noise.

The first effective approach for 3D electron diffraction was a combination of a stepwise rotation of the crystal with a small precession of the beam at each step.^104^ The crystal was then visualized and recentered after each tilt step, at the expense of an increase in electron dose. At the time the precession method was developed, electron diffraction data were always collected from crystals oriented along a crystallographic direction. Beam precession was used to reduce the dynamical scattering, which is enhanced in such conditions and makes the intensities deviate more strongly from the kinematic approximation.^99−101^ On the other hand, when coupled with 3D electron diffraction, beam precession is mostly used for smoothing the excitation error that affects intensities when the Ewald sphere does not cross the center of the reflection.^105^

Both 3D data collection strategies, either precession-assisted stepwise or by continuous rotation, reduce the effects of dynamical scattering and make the observed intensities better match with the kinematic approximation. This facilitates structure solution *ab initio*, for example by direct methods.^106^ Also model refinement is then possible with programs that X-ray crystallographers are familiar with, such as CRYSTALS, OLEX2, or SHELXL.^107−109^

### 4.4 Serial Electron Diffraction

Electrons interact strongly with matter, and time-resolution with TEMs reaches femtoseconds.^110,111^ Both properties make a single TEM as powerful as an X-ray free electron laser. A milestone for crystallography in this context was the structure determination of two proteins with serial electron diffraction (serialED).^42^ ED structures for small organic and inorganic compounds reach the same resolution limits as X-ray structures (0.55 Å in Figure 1F). This is very different for macromolecular structures: all ED structures of macromolecules published so far were about a factor of 2 worse in resolution compared with X-ray diffraction of the respective samples. Bücker et al.^42^ have been the first to demonstrate that ED of macromolecules can reach a similar resolution limit as macromolecular X-ray crystallography.

Serial crystallography for structure determination appears to be of little interest for small molecules. First, when crystals, or even crystalline powders, are available, they will be suitable for structure determination by 3D ED. Second, diffraction images of still crystals show much less Bragg peaks than for macromolecular compounds. This makes indexing and thus merging of the data extremely difficult. Time-resolution in the femtosecond regime of serial crystallography, however, would be interesting to study reactions, e.g. by stopped-flow experiments carried out in liquid-cell sample holders. The indexing problem could be solved with roto-serial crystallography.^112−114^ Roto-serial crystallography combines the diffraction with a very intense beam with a small, say 1°–5° rotation of the crystal. This is possible with TEMs, because unlike with samples for free electron lasers, the crystals are visible in the TEM and can thus be located, and the roto-shot can be prepared. Roto-serial ED appears promising especially for beam-sensitive phase mixtures of small-molecules, when combined with reliable clustering analysis. The software solution SerialRED might act as a prototype for this technique.^115^ The instrumental preconditions are already met by current TEMs.

### 4.5 Types of Grids and Sample Preparation

Crystals for electron diffraction are deposited on TEM grids. TEM grids are thin metal grids with a diameter of typically 3.05 mm. The metal gives mechanical stability to the grid and acts as conductor for captured electrons. The grids are commonly characterized by their mesh. A small mesh number, such as 150 or 200, results in large squares and thus increases the maximum available rotation range. Grids can be covered with a variety of thin layers, which support the sample and are electron-transparent. With less than 10 nm thickness, amorphous carbon is the thinnest material and therefore the preferable one for general electron diffraction applications. The stability can be increased with a layer of Formvar, at the cost of greater background noise.

There are special sample holders, with electron-transparent sample supports, allowing for a larger rotation range, even 360°.^116^ These special thin tips, however, are not produced commercially and have a large failure rate in production. Most data are therefore collected with normal TEM grids, coated with a nanometer-layer of continuous or lacy carbon. In case of low-symmetry space groups, complete data may not be collected from a single crystal. Either the crystal morphology favors various orientations of the crystals on the flat support layer or three-dimensional support grids can be used in order to achieve 100% complete data by merging data from several crystals. In order to create a three-dimensional support grid, the continuous carbon layer can be caused to coil by gently stroking it with a brush. Crystals stick with the coiled carbon layer, and therefore crystals in various orientations are conveniently found. This increases the chance to obtain high data completeness from only very few crystals, even in low-symmetry space groups.^117^ Moreover, continuous carbon has no visible features in the TEM imaging view. This makes orientation very difficult, and the alignment of the microscope for diffraction just off the crystal is impossible. Even coiled continuous carbon may not have features suitable for crystal centering. Holey and lacy carbon are much better suited for the purpose of crystal alignment and for orientation. At high magnification, even a single square of the grid is a huge area to scan. Therefore, orientation by the irregular patterns of lacy carbon is a great aid.

During data collection, the crystallographer is at risk of bias toward well diffracting crystals. This is particularly important, when mixtures of various phases or mixtures of different compounds are investigated. In such cases, conclusions about the sample can be supported with X-ray powder diffraction. A valuable approach in this context is the development of automated data collection, possibly combined with automated clustering of the sample, based on unit cell parameters.^61,115^ This technique can supplement X-ray powder diffraction and possibly find compounds in mixtures even if present in small trace amounts.

## 5 Applications

Chemists who apply X-ray crystallography for their research most likely would like to know whether 3D ED is a suitable technique for the compounds of their research. Generally speaking, whenever X-ray diffraction is part of the analytical toolbox, electron diffraction will also be useful. Gemmi et al.^6^ listed a number of applications where 3D ED was able to solve crystallographic problems that could not be addressed by X-ray. More recently, several studies exploited the fact that 3D ED can determine the single crystal structure when crystal size is the limiting factor for single crystal X-ray diffraction.^16,30,32,118^ A most impressive demonstration for organic compounds was published recently by Bruhn et al.^15^ 3D ED has been used to overcome local minima in crystal structure prediction.^119^ In our experience, sub-micrometer crystals of chemical compounds diffract to similar resolution with electron diffraction, as crystals tens of micrometers thick diffract with X-ray diffraction (Figure 1D and F). A crystal structure provides 3D coordinates of the atoms, that compose the compound, and their atomic displacement parameters, which indicate their thermal vibrations. When data quality is good, not only atom positions but also their element type can be determined.

The 3D coordinates from a crystal structure also provide information about distances of bonded and nonbonded contacts, about crystal packing, and about the space group. The space group has consequences, e.g. for electronic or optical properties. In materials science, the crystal structure helps with understanding crystal defects and describing the polymorphism of a material.^44^ In organic chemistry, a crystal structure provides qualitative proof for a synthesis pathway. In pharmacology, diffraction data helps to understand the kinematic and thermodynamic stability of a formulation.^120^ X-ray diffraction is also an established technique to determine the chirality.^121^

The large number of publications and reviews on 3D ED illustrate to what extend it complements X-ray crystallography in the above listed aspects, especially for those cases where single crystals cannot be grown to a size sufficiently large for X-ray diffraction.^4,6,7,43,122−124^ In particular, this complementarity also includes the determination of chirality with 3D ED.^18,125^ The following sections reflect our opinion, for which scientific applications of 3D ED will not only complement but actually extend X-ray crystallography.

### 5.1 Natural Products

A natural product is a chemical compound or substance produced by a living organism—that is, found in nature. [...] Within the field of organic chemistry, the definition of natural products is usually restricted to organic compounds isolated from natural sources that are produced by the pathways of primary or secondary metabolism. Within the field of medicinal chemistry, the definition is often further restricted to secondary metabolites.^126^

The interest in “natural products” lies in the interest to understand, improve, and exploit the diverse machinery available in nature. In many cases, their functionality involves modified standard amino-acids and circular peptides. The fields of interest include the health-section, with the research for antibacterial, antiviral, and anticancer drugs.^127−129^ They also include literally green sources of energy through the construction of metallochemical compounds that mimic the process of photosynthesis or the improvement of chemical reactions.^130^ Typically, natural products are only available in very small milligram amounts.^30^ This places a restriction on the possibilities of structure elucidation and makes electron diffraction a very attractive alternative to structure determination with X-rays or NMR. It is hard to imagine how little amounts are necessary for electron crystallography. A grain, just about visible with the bare eye, can contain hundreds or thousands of single crystals. This explains the interest in 3D ED for structure determination for cases where the amount of material is very limited, e.g. due to difficulties in purification.

That said, one has to bear in mind that sample preparation for 3D ED takes some experience. The main difficulty is the preparation of a TEM grid with the right density of particles. Too many particles result in agglomeration of crystals and make it difficult to find single crystals. This is certainly true for natural products that can often be quite sticky. Too few particles make it very cumbersome to find any crystals at all. With some experience in grid preparation, testing less than 5–10 different grids should lead to success.^5^

It is convenient to allow for a certain size distribution of particles. Small crystals, that are suitable for data collection, are often close to larger clusters on the sample grid. These large clusters can be found when scanning the TEM grid at low magnification. The hit score can be improved with a TEM equipped with a STEM unit.^65^ The dose required for STEM imaging is much lower than for a real image at the same resolution, which reduces radiation damage in the case of very sensitive samples.^8,87,122^ In addition to the reduced dose, the STEM imaging takes place in the diffraction mode of the TEM, so that no switching of the lens system is required before data collection.

### 5.2 Charges: Oxidation States, Noninnocent Ligands

In terms of structure determination, electron diffraction is quite analogous to X-ray diffraction. The result is a map, and the chemical model is an interpretation of the map. The atoms are located at the map peaks. Model building is guided through the difference map. Physically, electron diffraction occurs through the interaction of the electron beam with the local electrostatic potential inside the crystal. This causes an interesting feature for electron diffraction which is different from X-ray diffraction: the scattering factors of ions diverge at low resolution. For cations, the scattering factor diverges to infinity; for anions, it diverges to minus infinity.^131,132^

**Figure 2:**
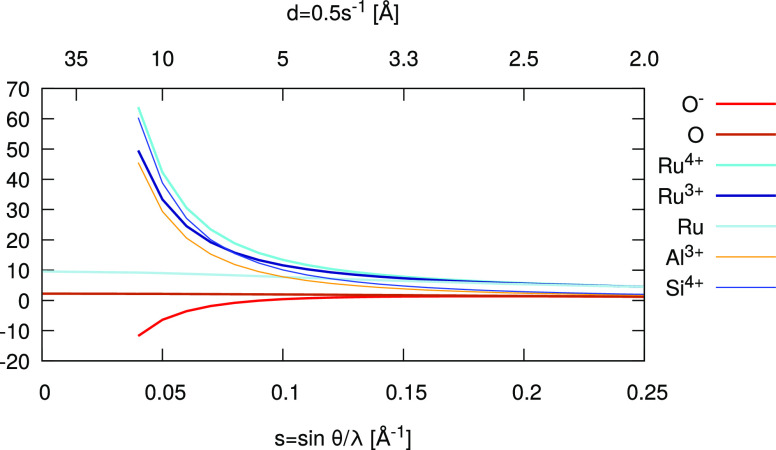
Electron scattering factors for ions and noncharged elements. The graph illustrates the divergence of ionic scattering factors to ±*∞* for *s* → 0 Å^–1^.

Figure 2 shows the theoretical scattering factors for a few selected elements. The scattering factor for the negative *O*^–^ ion diverges to minus infinity. The scattering factors of different oxidations states are rather different and allow for their differentiation. Figure 2 includes *Ru*, *Ru*^3+^, and *Ru*^4+^ as an example. In order to demonstrate that this difference is strong enough to differentiate between these oxidation states, the scattering curves for *Al*^3+^ and *Si*^4+^ are also shown. These two elements were recently differentiated with electron diffraction in the case of two aluminosilicates.^35^ It is worth noting that for aluminosilicates, neither element is expected to be at their full oxidation state and the actual difference in their scattering power should be less than the difference between their theoretical scattering curves. As Figure 2 illustrates, electron diffraction is sensitive to the oxidation states especially at low-resolution data. Large unit cells result in more data points at low resolution and may make it easier to differentiate ionic states.^131^ The lowest resolution reflection of albite, one of the samples where silicon was differentiated from aluminum,^35^ is only at *d* = 6.35 Å, i.e. sin θ/λ = 0.0787 1/Å. Therefore, the use of electron diffraction to probe for ionic states not only applies to proteins, but indeed to most chemical compounds, either organic or inorganic. In fact, the electrostatic potential in salts can be determined quantitatively with electron diffraction data.^133,134^

This characteristic of electron diffraction becomes interesting in cases where the oxidation states are not purely ionic and where they are not easily assigned to individual atoms. Such complex situations arise when both cation and anion can have multiple oxidation states, e.g. when noninnocent ligands are studied. A “non-innocent ligand is a ligand in a metal complex, where the oxidation state is not clear”.^135^ Noninnocent ligands are only one example where 3D ED may become the tool of choice. In many chemical systems, the electronic state escapes simulation studies, while charge density studies with X-ray diffraction require such high-resolution data that may not be available.^136,137^

### 5.3 Twinning

Ideally, diffraction data are collected from only a single crystal. Twinning refers to a data set from two or more crystal lattices. This can occur on the domain level; that is, a seemingly single crystal is composed of unit cells which are not related to pure translation. It can also occur at the macroscopic level, when two crystals are attached to one another.^138^ Twinning may introduce space group ambiguities or destabilize the convergence of the refinement procedure. In case of an arbitrary twin law, the integration program can pick out the major lattice and ignore the contribution of smaller lattices. In case of overlapping reflections, however, this leads to corruption of the observed intensity. Advanced programs can deconvolute several lattices during data processing. The algorithms for the treatment of twinned data do not rely on the radiation source and therefore are also applicable to 3D ED data.^139,140^ In this context, 3D ED has a great advantage compared with X-ray diffraction: Since 3D ED can collect data from domains as small as the beam diameter, i.e. down to 30 nm, it offers the chance of getting single crystal data from crystals, that macroscopically appear twinned, but microscopically are single.^44,141,142^ At high magnification, individual domains may become apparent, and data can be collected from single domains which are too small to be seen with the optical microscope.

**Figure 3:**
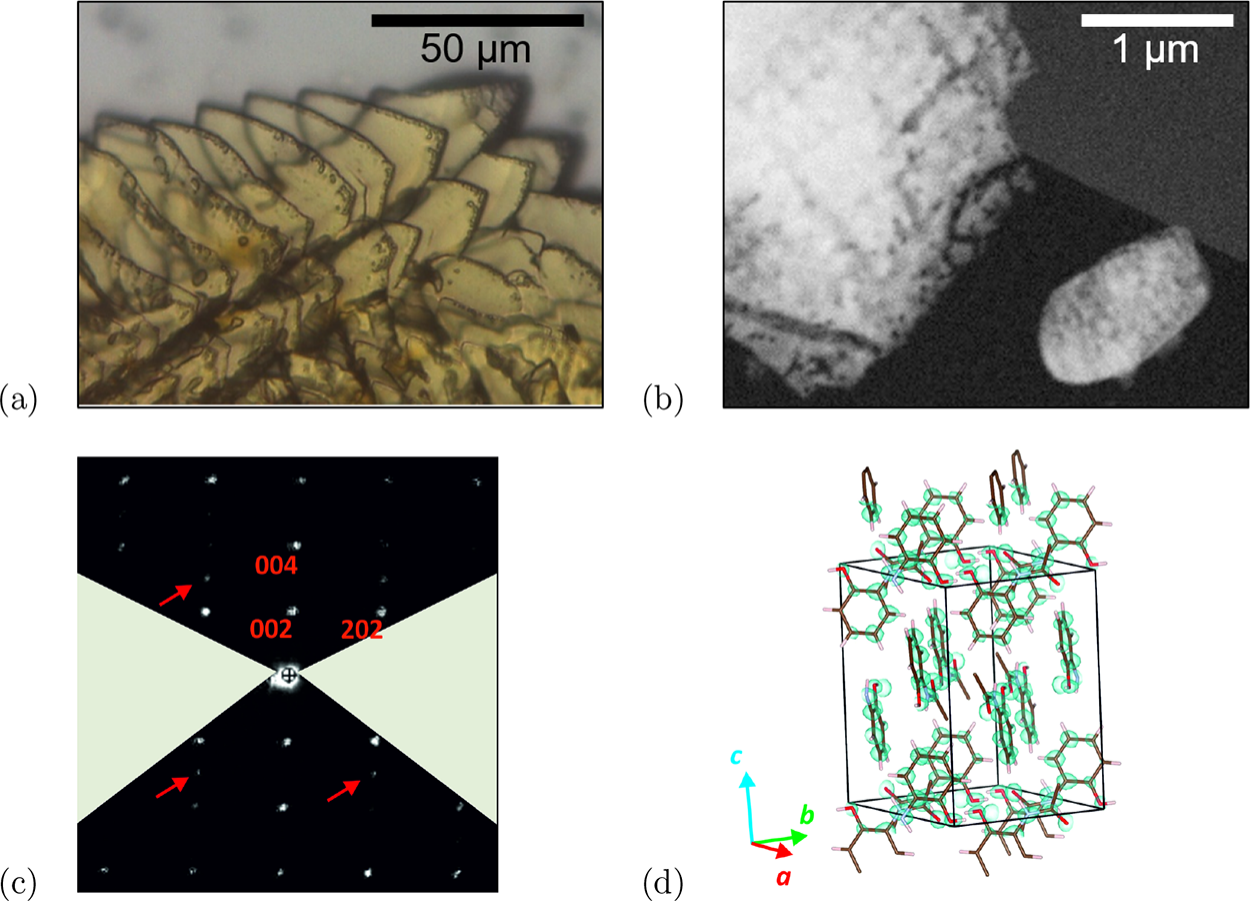
(a) Optical microscope image of a typical orthocetamol polycrystalline aggregate. (b) Dark-field STEM image of orthocetamol fragments. The fragment on the bottom right corner has an optimal size for 3D ED data collection. (c) Reconstructed *h*0*l* slice of reciprocal space extracted from a 3D ED data collection. Extinctions due to *C*-centering are clearly visible, while extinctions due to the *c*-glide plane are partially violated by reflections coming from a twin domain. (d) Orthocetamol structure, made of alternating layers of orthocetamol chains oriented along [110] and [−110].

This has been recently achieved for the structure of orthocetamol (Figure 3). Light microscopy shows strongly intergrown crystals, which could not be singularly sampled by X-ray diffraction^140^ (Figure 3(a)). Indeed, orthocetamol domains are often smaller than 100 nm and only using electron diffraction it was possible to obtain structural data from volumes of sample that are mostly single crystal (Figure 3(b)). The structure of orthocetamol is monoclinic, space group *C*2/*c*, and is characterized by two cell parameters with relatively close lengths (*a* = 10.5612 Å and *b* = 10.3856 Å) and by a β-angle not far from 90°. Orthocetamol tends to form twin domains after a rotation of 90° around the [001] axis. However, the occurrence of diffuse scattering makes it difficult to recognize the splitting of reflections originated by two twin domains (Figure 3(c)). The overlap of the two lattices results also in the apparent violation of the extinction rule related with the *c*-glide plane, and therefore, the structure was first assigned to the tetragonal systems. Still, all structural solution attempts failed, until the actual monoclinic symmetry was recognized and imposed. The *ab initio* model was later refined with SHELXL, and it was found that the secondary twin domain accounts for 34% of the diffraction intensities (Figure 3(d)).

## 6 Conclusions and Outlook

Less than two decades after the first milestone experiments^143^ and only three years after its nomination as a breakthrough technology in Science,^144^ 3D ED has become a routine technique for chemical analysis.^6,15^ It complements X-ray crystallography where crystal size matters. It also extends X-ray crystallography for greater sensitivity to chirality,^18^ for the detection of light atoms in the presence of heavy atoms,^17^ and 3D ED is more sensitive to charge, including partial charges. Especially the latter property has not been fully exploited yet, and we are confident that future development will open new possibilities for chemists and materials scientists. For its exciting future to happen, broader acceptance and application of the technology will be essential. We previously elaborated that 3D ED is best practised with a crystallographer’s mind.^22^ In this review we explain the required instrumentation. For general purposes, we recommend a *LaB*_6_ TEM equipped with a hybrid pixel detector. The maximum available energy for a *LaB*_6_ TEM is currently 200 keV. With costs comparable to a laboratory X-ray diffractometer, every X-ray facility is now equipped with the knowledge to complement their instruments with 3D electron diffraction.

## References

[ref1] Gruene T.; Wennmacher J. T. C.; Zaubitzer C.; Holstein J. J.; Heidler J.; Fecteau Lefebvre A.; De Carlo S.; Müller E.; Goldie K. N.; Regeni I.; et al. Angew. Chem., Int. Ed. 2018, 57, 16313–16317. 10.1002/anie.201811318. PMC6468266 30325568

[ref2] Jones C. G.; Martynowycz M. W.; Hattne J.; Fulton T. J.; Stoltz B. M.; Rodriguez J. A.; Nelson H. M.; Gonen T. ACS Cent. Sci. 2018, 4, 1587–1592. 10.1021/acscentsci.8b00760. 30555912 PMC6276044

[ref3] Warren M. Nature 2018, 563, 16–17. 10.1038/d41586-018-07213-3. 30377327

[ref4] Nannenga B. L. Struct. Dyn. 2020, 7, 014304 10.1063/1.5128226. 32071929 PMC7018523

[ref5] Clabbers M. T.; Xu H. Drug Discovery Today: Technol. 2020, 10.1016/j.ddtec.2020.12.002. 34895659

[ref6] Gemmi M.; Mugnaioli E.; Gorelik T. E.; Kolb U.; Palatinus L.; Boullay P.; Hovmöller S.; Abrahams J. P. ACS Cent. Sci. 2019, 5, 1315–1329. 10.1021/acscentsci.9b00394. 31482114 PMC6716134

[ref7] Huang Z.; Grape E. S.; Li J.; Inge A. K.; Zou X. Coord. Chem. Rev. 2021, 427, 213583 10.1016/j.ccr.2020.213583.

[ref8] Kolb U.; Gorelik T.; Kübel C.; Otten M. T.; Hubert D. Ultramicroscopy 2007, 107, 507–513. 10.1016/j.ultramic.2006.10.007. 17234347

[ref9] Nannenga B. L.; Shi D.; Leslie A. G. W.; Gonen T. Nat. Methods 2014, 11, 927–930. 10.1038/nmeth.3043. 25086503 PMC4149488

[ref10] Zhang D.; Oleynikov P.; Hovmöller S.; Zou X. Z. Kristallogr. - Cryst. Mater. 2010, 225, 94–102. 10.1524/zkri.2010.1202.

[ref11] Wang Y.; Takki S.; Cheung O.; Xu H.; Wan W.; Öhrström L.; Inge A. K. Chem. Commun. 2017, 53, 7018–7021. 10.1039/C7CC03180G. 28613325

[ref12] Gemmi M.; Oleynikov P. Z. Z. Kristallogr. - Cryst. Mater. 2013, 228, 51–58. 10.1524/zkri.2013.1559.

[ref13] Gemmi M.; La Placa M. G. I.; Galanis A. S.; Rauch E. F.; Nicolopoulos S. J. J. Appl. Crystallogr. 2015, 48, 718–727. 10.1107/S1600576715004604.

[ref14] Boullay P.; Palatinus L.; Barrier N. Inorg. Chem. 2013, 52, 6127–6135. 10.1021/ic400529s. 23634789

[ref15] Bruhn J. F.; Scapin G.; Cheng A.; Mercado B. Q.; Waterman D. G.; Ganesh T.; Dallakyan S.; Read B. N.; Nieusma T.; Lucier K. W.; et al. Front. Mol. Biosci. 2021, 10.3389/fmolb.2021.648603. PMC8313502 34327213

[ref16] Steciuk G.; Schäf O.; Tortet L.; Pizzala H.; Palatinus L.; Hornfeck W.; Paillaud J.-L. Eur. J. Inorg. Chem. 2021, 2021, 628–638. 10.1002/ejic.202000939.

[ref17] Palatinus L.; Brázda P.; Boullay P.; Perez O.; Klementová M.; Petit S.; Eigner V.; Zaarour M.; Mintova S. Science 2017, 355, 166–169. 10.1126/science.aak9652. 28082587

[ref18] Brázda P.; Palatinus L.; Babor M. Science 2019, 364, 667–669. 10.1126/science.aaw2560. 31097664

[ref19] Authier A. Dynamical Theory of X-Ray Diffraction; IUCr Monographs on Crystallography; Oxford University Press Inc.: New York, 2001.

[ref20] Palatinus L.; Petříčk V.; Corrêa C. A. Acta Crystallogr., Sect. A: Found. Adv. 2015, A71, 235–244. 10.1107/S2053273315001266. 25727873

[ref21] Palatinus L.; Corrêa C. A.; Steciuk G.; Jacob D.; Roussel P.; Boullay P.; Klementová M.; Gemmi M.; Kopeček J.; Domeneghetti M. C.; et al. Acta Crystallogr., Sect. B: Struct. Sci., Cryst. Eng. Mater. 2015, B71, 740–751. 10.1107/S2052520615017023. 26634732

[ref22] Gruene T.; Holstein J. J.; Clever G. H.; Keppler B. Nat. Rev. Chem. 2021, 5, 660–668. 10.1038/s41570-021-00302-4. 37118416

[ref23] Palatinus L.; Brázda P.; Jelínek M.; Hrdá J.; Steciuk G.; Klementová M. Acta Crystallogr., Sect. B: Struct. Sci., Cryst. Eng. Mater. 2019, B75, 512–522. 10.1107/S2052520619007534. 32830709

[ref24] Groom C. R.; Bruno I. J.; Lightfoot M. P.; Ward S. C. Acta Crystallogr., Sect. B: Struct. Sci., Cryst. Eng. Mater. 2016, B72, 171–179. 10.1107/S2052520616003954. PMC4822653 27048719

[ref25] Johnson N. Electron Diffraction Data in the CSD; 2020.

[ref26] Steciuk G.; Majzlan J.; Plášil J. IUCrJ 2021, 8, 116–123. 10.1107/S2052252520015626. PMC7793002 33520247

[ref27] Debost M.; Klar P. B.; Barrier N.; Clatworthy E. B.; Grand J.; Laine F.; Brázda P.; Palatinus L.; Nesterenko N.; Boullay P.; et al. Angew. Chem., Int. Ed. 2020, 59, 23491 10.1002/anie.202009397. 32902156

[ref28] Vergara S.; Lukes D. A.; Martynowycz M. W.; Santiago U.; Plascencia-Villa G.; Weiss S. C.; de la Cruz M. J.; Black D. M.; Alvarez M. M.; López-Lozano X.; et al. J. Phys. Chem. Lett. 2017, 8, 5523–5530. 10.1021/acs.jpclett.7b02621. 29072840 PMC5769702

[ref29] Jones C. G.; Asay M.; Kim L. J.; Kleinsasser J. F.; Saha A.; Fulton T. J.; Berkley K. R.; Cascio D.; Malyutin A. G.; Conley M. P.; et al. ACS Cent. Sci. 2019, 5, 1507–1513. 10.1021/acscentsci.9b00403. 31572777 PMC6764211

[ref30] Kim L. J.; Ohashi M.; Zhang Z.; Tan D.; Asay M.; Cascio D.; Rodriguez J. A.; Tang Y.; Nelson H. M. Nat. Chem. Biol. 2021, 17, 872–877. 10.1038/s41589-021-00834-2. 34312563 PMC8447837

[ref31] Richards L. S.; Millán C.; Miao J.; Martynowycz M. W.; Sawaya M. R.; Gonen T.; Borges R. J.; Usón I.; Rodriguez J. A. Acta Crystallogr. 2020, D76, 703–712. 10.1107/S2059798320008049. PMC7397493 32744252

[ref32] Broadhurst E. T.; Xu H.; Clabbers M. T. B.; Lightowler M.; Nudelman F.; Zou X.; Parsons S. IUCrJ 2020, 7, 5–9. 10.1107/S2052252519016105. PMC6949601 31949899

[ref33] Cichocka M. O.; Liang Z.; Feng D.; Back S.; Siahrostami S.; Wang X.; Samperisi L.; Sun Y.; Xu H.; Hedin N.; et al. J. Am. Chem. Soc. 2020, 142, 15386–15395. 10.1021/jacs.0c06329. 32786758 PMC7498152

[ref34] Xu H.; Lebrette H.; Clabbers M. T. B.; Zhao J.; Griese J. J.; Zou X.; Högbom M. Sci. Adv. 2019, 5, 10.1126/sciadv.aax4621. PMC6685719 31457106

[ref35] Fröjdh E.; Wennmacher J. T. C.; Rzepka P.; Mozzanica A.; Redford S.; Schmitt B.; van Bokhoven J. A.; Gruene T. Crystals 2020, 10, 1148 10.3390/cryst10121148.

[ref36] Bruhn J.; Cheng A. Progesterone Electron Diffraction (MicroED) Datasets - Glacios TEM with a CETA-D (accessed 28/12/2020).

[ref37] Lu H.; Nakamuro T.; Yamashita K.; Yanagisawa H.; Nureki O.; Kikkawa M.; Gao H.; Tian J.; Shang R.; Nakamura E. J. Am. Chem. Soc. 2020, 142, 18990–18996. 10.1021/jacs.0c10337. 33089998

[ref38] Van Genderen E.; Clabbers M. T. B.; Das P. P.; Stewart A.; Nederlof I.; Barentsen K. C.; Portillo Q.; Pannu N. S.; Nicolopoulos S.; Gruene T.; et al. Acta Crystallogr., Sect. A: Found. Adv. 2016, A72, 236–242. 10.1107/S2053273315022500. PMC4770873 26919375

[ref39] Matz J. M.; Drepper B.; Blum T. B.; van Genderen E.; Burrell A.; Martin P.; Stach T.; Collinson L. M.; Abrahams J. P.; Matuschewski K.; et al. Proc. Natl. Acad. Sci. U. S. A. 2020, 117, 16546–16556. 10.1073/pnas.2001153117. 32601225 PMC7368307

[ref40] Takaba K.; Maki-Yonekura S.; Inoue S.; Hasegawa T.; Yonekura K. Front. Mol. Biosci. 2021, 7, 440 10.3389/fmolb.2020.612226. PMC7814344 33469549

[ref41] Takaba K.; S M.-Y.; Yonekura K. J. J. Struct. Biol. 2020, 211, 107549 10.1016/j.jsb.2020.107549. 32544623

[ref42] Bücker R.; Hogan-Lamarre P.; Mehrabi P.; Schulz E. C.; Bultema L. A.; Gevorkov Y.; Brehm W.; Yefanov O.; Oberthür D.; Kassier G. H.; et al. Nat. Commun. 2020, 11, 996 10.1038/s41467-020-14793-0. 32081905 PMC7035385

[ref43] Lanza A.; Margheritis E.; Mugnaioli E.; Cappello V.; Garau G.; Gemmi M. IUCrJ 2019, 6, 178–188. 10.1107/S2052252518017657. PMC6400191 30867915

[ref44] Mugnaioli E.; Bonaccorsi E.; Lanza A. E.; Elkaim E.; Diez-Gómez V.; Sobrados I.; Gemmi M.; Gregorkiewitz M. IUCrJ 2020, 7, 1070–1083. 10.1107/S2052252520012270. PMC7642771 33209318

[ref45] Duyvesteyn H. M. E.; Kotecha A.; Ginn H. M.; Hecksel C. W.; Beale E. V.; de Haas F.; Evans G.; Zhang P.; Chiu W.; Stuart D. I. Proc. Natl. Acad. Sci. U. S. A. 2018, 115, 9569 10.1073/pnas.1809978115. 30171169 PMC6156647

[ref46] Beale E. V.; Waterman D. G.; Hecksel C.; van Rooyen J.; Gilchrist J. B.; Parkhurst J. M.; de Haas F.; Buijsse B.; Evans G.; Zhang P. Front. Mol. Biosci. 2020, 7, 10.3389/fmolb.2020.00179. PMC7417479 32850967

[ref47] Sakamoto Y.; Zhao H.; Gies H.; Yamamoto K.; Kolb U.; Ikeda T. Dalton Trans 2020, 49, 12960–12969. 10.1039/D0DT02290J. 32936162

[ref48] Marler B.; Krysiak Y.; Kolb U.; Grafweg C.; Gies H. Microporous Mesoporous Mater. 2020, 296, 109981 10.1016/j.micromeso.2019.109981.

[ref49] Gonano B.; Bréard Y.; Pelloquin D.; Caignaert V.; Perez O.; Pautrat A.; Boullay P.; Bazin P.; Le Breton J.-M. Inorg. Chem. 2017, 56, 15241–15250. 10.1021/acs.inorgchem.7b02572. 29215869

[ref50] Karakulina O. M.; Demortière A.; Dachraoui W.; Abakumov A. M.; Hadermann J. Nano Lett. 2018, 18, 6286–6291. 10.1021/acs.nanolett.8b02436. 30193062

[ref51] Kodjikian S.; Klein H. Ultramicroscopy 2019, 200, 12–19. 10.1016/j.ultramic.2019.02.010. 30797182

[ref52] Grey I. E.; Yoruk E.; Kodjikian S.; Klein H.; Bougerol C.; Brand H. E.; Bordet P.; Mumme W. G.; Favreau G.; Mills S. Mineral. Mag. 2020, 84, 608–615. 10.1180/mgm.2020.52.

[ref53] Duran E. C.; Eggeman A. S. J. Solid State Chem. 2021, 293, 121795 10.1016/j.jssc.2020.121795.

[ref54] Gleason P. R.; Nannenga B. L.; Mills J. H. Front. Mol. Biosci. 2021, 7, 461 10.3389/fmolb.2020.609999. PMC7821094 33490105

[ref55] Rondeau B.; Devouard B.; Jacob D.; Roussel P.; Stephant N.; Boulet C.; Mollé V.; Corre M.; Fritsch E.; Ferraris C.; et al. Eur. J. Mineral. 2019, 31, 379–388. 10.1127/ejm/2019/0031-2817.

[ref56] Vainshtein B. K. Structure Analysis by electron Diffraction; Pergamon Press: Oxford, 1964.

[ref57] ELDICO Scientific, the Electron Diffraction Company. https://www.eldico-scientific.com/ (accessed 05/07/2021).

[ref58] Rigaku, XtaLAB Synergy-ED. https://www.rigaku.com/products/crystallography/synergy-ed (accessed 24/06/2021).

[ref59] Cichocka M. O.; Ångström J.; Wang B.; Zou X.; Smeets S. J. Appl. Crystallogr. 2018, 51, 1652–1661. 10.1107/S1600576718015145. 30546290 PMC6276279

[ref60] Roslova M.; Smeets S.; Wang B.; Thersleff T.; Xu H.; Zou X. J. Appl. Crystallogr. 2020, 53, 1217–1224. 10.1107/S1600576720009590. 33117109 PMC7534539

[ref61] Yonekura K.; Ishikawa T.; Maki-Yonekura S. J. Struct. Biol. 2019, 206, 243–253. 10.1016/j.jsb.2019.03.009. 30928615

[ref62] Plana-Ruiz S.; Krysiak Y.; Portillo J.; Alig E.; Estradé S.; Peiró F.; Kolb U. Ultramicroscopy 2020, 211, 112951 10.1016/j.ultramic.2020.112951. 32036199

[ref63] De la Cruz M. J.; Martynowycz M. W.; Hattne J.; Gonen T. Ultramicroscopy 2019, 201, 77–80. 10.1016/j.ultramic.2019.03.009. 30986656 PMC6752703

[ref64] Mastronarde D. N. J. Struct. Biol. 2005, 152, 36–51. 10.1016/j.jsb.2005.07.007. 16182563

[ref65] Reimer L.; Kohl H. Transmission Electron Microscopy, Physics of Image Formation; Springer-Verlag: New York, 2008.

[ref66] Bilderback D. H. Nucl. Instrum. Methods Phys. Res. 1982, 195, 67–72. 10.1016/0029-554X(82)90759-5.

[ref67] Yonekura K.; Maki-Yonekura S.; Namba K. Biophys. J. 2002, 82, 2784–2797. 10.1016/S0006-3495(02)75619-1. 11964264 PMC1302066

[ref68] Parkhurst J. M.; Winter G.; Waterman D. G.; Fuentes-Montero L.; Gildea R. J.; Murshudov G. N.; Evans G. J. Appl. Crystallogr. 2016, 49, 1912–1921. 10.1107/S1600576716013595. 27980508 PMC5139990

[ref69] Latychevskaia T.; Abrahams J. P. Acta Crystallogr., Sect. B: Struct. Sci., Cryst. Eng. Mater. 2019, B75, 523–531. 10.1107/S2052520619009661. PMC6690131 32830710

[ref70] Llovet X. J. Phys. Chem. Ref. Data 2014, 43, 013102.

[ref71] J. ICRU 2014, 14, 111. 10.1093/jicru_ndx002

[ref72] McMullan G.; Chen S.; Henderson R.; Faruqi A. Ultramicroscopy 2009, 109, 1126–1143. 10.1016/j.ultramic.2009.04.002. 19497671 PMC2864625

[ref73] Tinti G.; Fröjdh E.; van Genderen E.; Gruene T.; Schmitt B.; de Winter D. A. M.; Weckhuysen B. M.; Abrahams J. P. IUCrJ 2018, 5, 190–199. 10.1107/S2052252518000945. PMC5947724 29765609

[ref74] Naydenova K.; McMullan G.; Peet M. J.; Lee Y.; Edwards P. C.; Chen S.; Leahy E.; Scotcher S.; Henderson R.; Russo C. IUCrJ 2019, 6, 1086–1098. 10.1107/S2052252519012612. PMC6830209 31709064

[ref75] Henderson R. Q. Q. Rev. Biophys. 1995, 28, 171–193. 10.1017/S003358350000305X. 7568675

[ref76] Zuo J. M.; Spence J. C. H. Advanced Transmission Electron Microscopy, Imaging and Diffraction in Nanoscience; Springer Science+Business Media: New York, 2017: 2016.

[ref77] Carter C. B.; Williams D. B. Transmission Electron Microscopy, Diffraction, Imaging, and Spectrometry; Springer International Publishing: Switzerland, 2016.

[ref78] Egerton R. F. Microsc. Res. Tech. 2012, 75, 1550–1556. 10.1002/jemt.22099. 22807142

[ref79] Garman E. F. Acta Crystallogr., Sect. D: Biol. Crystallogr. 2010, D66, 339–351. 10.1107/S0907444910008656. PMC2852297 20382986

[ref80] Christensen J.; Horton P. N.; Bury C. S.; Dickerson J. L.; Taberman H.; Garman E. F.; Coles S. J. IUCrJ 2019, 6, 703–713. 10.1107/S2052252519006948. PMC6608633 31316814

[ref81] Hattne J.; Martynowycz M. W.; Penczek P. A.; Gonen T. IUCrJ 2019, 6, 921–926. 10.1107/S2052252519010583. PMC6760445 31576224

[ref82] Tietz Video and Image Processing Systems. https://www.tvips.com/ (accessed 09/09/2021).

[ref83] ThermoFisher Scientific. https://www.thermofisher.com/ (accessed 09/09/2021).

[ref84] Gatan. https://www.gatan.com/ (accessed 09/09/2021).

[ref85] Direct Electron. https://www.directelectron.com/ (accessed 09/09/2021).

[ref86] Broennimann C.; Eikenberry E. F.; Henrich B.; Horisberger R.; Huelsen G.; Pohl E.; Schmitt B.; Schulze-Briese C.; Suzuki M.; Tomizaki T.; et al. J. Synchrotron Radiat. 2006, 13, 120–130. 10.1107/S0909049505038665. 16495612

[ref87] Heidler J.; Pantelic R.; Wennmacher J. T. C.; Zaubitzer C.; Fecteau-Lefebvre A.; Goldie K. N.; Müller E.; Holstein J. J.; van Genderen E.; De Carlo S.; et al. Acta Crystallogr. 2019, D75, 458–466. 10.1107/S2059798319003942. PMC6503764 31063148

[ref88] ASI Amsterdam Scientific Instruments. https://www.amscins.com (accessed 13/07/2021).

[ref89] DECTRIS Ltd. https://www.dectris.com (accessed 13/07/2021).

[ref90] Quantum Detectors. https://quantumdetectors.com/ (accessed 09/09/2021).

[ref91] Rigaku. https://rigaku.com (accessed 13/07/2021).

[ref92] X-Spectrum. https://x-spectrum.de/ (accessed 09/09/2021).

[ref93] French S.; Wilson K. Acta Crystallogr., Sect. A: Cryst. Phys., Diffr., Theor. Gen. Crystallogr. 1978, A34, 517–525. 10.1107/S0567739478001114.

[ref94] Hattne J.; Shi D.; de la Cruz M. J.; Reyes F. E.; Gonen T. J. Appl. Crystallogr. 2016, 49, 1029–1034. 10.1107/S1600576716007196. 27275145 PMC4886988

[ref95] Waterman D. G. Investigate pedestal over a narrow range from 0 to −30. https://github.com/dagewa/wedged-lamellae (accessed 13/07/2021).

[ref96] Dauter Z. Acta Crystallogr., Sect. D: Biol. Crystallogr. 2010, D66, 389–392. 10.1107/S0907444909038578. PMC2852303 20382992

[ref97] Jacques Dubochet – Facts. https://www.nobelprize.org/prizes/chemistry/2017/dubochet/facts (accessed 24/02/2021).

[ref98] Kashin A. S.; Ananikov V. P. Nat. Rev. Chem. 2019, 3, 624–637. 10.1038/s41570-019-0133-z.

[ref99] Vincent R.; Midgley P. A. Ultramicroscopy 1994, 53, 271–282. 10.1016/0304-3991(94)90039-6.

[ref100] White T. A. Structure Solution Using Precession Electron Diffraction and Diffraction Tomography; PhD, University of Cambridge, 2009.

[ref101] Eggeman A. S.; Midgley P. A. In Advances in Imaging and Electron Physics; Advances in Imaging and Electron Physics, Vol. 170; Elsevier: 2012; pp 1–63.

[ref102] The rotation method in crystallography; Arndt W., Wonacott A., Eds.; Amsterdam a.o.: North-Holland, 1977.

[ref103] Brönnimann C.; Eikenberry E. F.; Horisberger R.; Hülsen G.; Schmitt B.; Schulze-Briese C.; Tomizaki T. Nucl. Instrum. Methods Phys. Res., Sect. A 2003, 510, 24–28. 10.1016/S0168-9002(03)01673-5.

[ref104] Mugnaioli E.; Gorelik T.; Kolb U. Ultramicroscopy 2009, 109, 758–765. 10.1016/j.ultramic.2009.01.011. 19269095

[ref105] Palatinus L.; Jacob D.; Cuvillier P.; Klementová M.; Sinkler W.; Marks L. D. Acta Crystallogr., Sect. A: Found. Crystallogr. 2013, A69, 171–188. 10.1107/S010876731204946X. 23403968

[ref106] Dorset D. L. Acta Crystallogr., Sect. A: Found. Crystallogr. 1998, A54, 750–757. 10.1107/S0108767398006722. 9859193

[ref107] Betteridge P. W.; Carruthers J. R.; Cooper R. I.; Prout K.; Watkin D. J. Appl. Crystallogr. 2003, 36, 1487 10.1107/S0021889803021800.

[ref108] Dolomanov O. V.; Blake A. J.; Champness N. R.; Schröder M. J. Appl. Crystallogr. 2003, 36, 1283–1284. 10.1107/S0021889803015267.

[ref109] Sheldrick G. M. Acta Crystallogr. 2015, C71, 3–8. 10.1107/S2053229614024218. PMC4283466 25537383

[ref110] YANG J.; YOSHIDA Y.; SHIBATA H. Electron. Commun. Jpn. 2015, 98, 50–57. 10.1002/ecj.11763.

[ref111] Feist A.; Bach N.; Rubiano da Silva N.; Danz T.; Möller M.; Priebe K. E.; Domröse T.; Gatzmann J. G.; Rost S.; Schauss J.; et al. Ultramicroscopy 2017, 176, 63–73. 10.1016/j.ultramic.2016.12.005. 28139341

[ref112] Gati C.; Bourenkov G.; Klinge M.; Rehders D.; Stellato F.; Oberthür D.; Yefanov O.; Sommer B. P.; Mogk S.; Duszenko M.; et al. IUCrJ 2014, 1, 87–94. 10.1107/S2052252513033939. PMC4062088 25075324

[ref113] Hasegawa K.; Yamashita K.; Murai T.; Nuemket N.; Hirata K.; Ueno G.; Ago H.; Nakatsu T.; Kumasaka T.; Yamamoto M. J. Synchrotron Radiat. 2017, 24, 29–41. 10.1107/S1600577516016362. 28009544 PMC5182019

[ref114] Wierman J. L.; Paré-Labrosse O.; Sarracini A.; Besaw J. E.; Cook M. J.; Oghbaey S.; Daoud H.; Mehrabi P.; Kriksunov I.; Kuo A.; et al. IUCrJ 2019, 6, 305–316. 10.1107/S2052252519001453. PMC6400179 30867928

[ref115] Wang B.; Zou X.; Smeets S. IUCrJ 2019, 6, 854–867. 10.1107/S2052252519007681. PMC6760450 31576219

[ref116] Palmer C. M.; Löwe J. Ultramicroscopy 2014, 137, 20–29. 10.1016/j.ultramic.2013.10.016. 24275523 PMC4054515

[ref117] Wennmacher J. T. C.; Zaubitzer C.; Li T.; Bahk Y. K.; Wang J.; van Bokhoven J. A.; Gruene T. Nat. Commun. 2019, 10, 3316 10.1038/s41467-019-11326-2. 31346178 PMC6658500

[ref118] Andrusenko I.; Potticary J.; Hall S. R.; Gemmi M. Acta Crystallogr., Sect. B: Struct. Sci., Cryst. Eng. Mater. 2020, B76, 1036–1044. 10.1107/S2052520620012779. 33289715

[ref119] Cui P.; Svensson Grape E.; Spackman P. R.; Wu Y.; Clowes R.; Day G. M.; Inge A. K.; Little M. A.; Cooper A. I. J. Am. Chem. Soc. 2020, 142, 12743–12750. 10.1021/jacs.0c04885. 32597187 PMC7467715

[ref120] Ng A.; Lai C.; Dabros M.; Gao Q. J. Pharm. Sci. 2014, 103, 3423–3431. 10.1002/jps.24135. 25252084

[ref121] Derewenda Z. S. Acta Crystallogr., Sect. A: Found. Crystallogr. 2008, A64, 246–258. 10.1107/S0108767307054293. 18156689

[ref122] Gemmi M.; Lanza A. E. Acta Crystallogr., Sect. B: Struct. Sci., Cryst. Eng. Mater. 2019, B75, 495–504. 10.1107/S2052520619007510. 32830707

[ref123] Kolb U.; Krysiak Y.; Plana-Ruiz S. Acta Crystallogr., Sect. B: Struct. Sci., Cryst. Eng. Mater. 2019, B75, 463–474. 10.1107/S2052520619006711. PMC6690130 32830704

[ref124] Hadermann J., Palatinus L., Eds. Acta Crystallogr. B66 (2019): Special issue on electron crystallography. 10.1107/S2052520619010783 32830703

[ref125] Ma Y.; Oleynikov Pand Terasaki O. Nat. Mater. 2017, 16, 755–759. 10.1038/nmat4890. 28459446

[ref126] Natural Products. https://en.wikipedia.org/wiki/Natural_product (accessed 06/02/2021).

[ref127] Rossiter S. E.; Fletcher M. H.; Wuest W. M. Chem. Rev. 2017, 117, 12415–12474. 10.1021/acs.chemrev.7b00283. 28953368 PMC5869711

[ref128] Antonio A. d. S.; Wiedemann L. S. M.; Veiga-Junior V. F. RSC Adv. 2020, 10, 23379–23393. 10.1039/D0RA03774E. PMC9122563 35693131

[ref129] Cragg G. M.; Pezzuto J. M. Medical Principles and Practice 2016, 25, 41–59. 10.1159/000443404. 26679767 PMC5588531

[ref130] Dalle K.; Gruene T.; Dechert S.; Demeshko S.; Meyer F. J. Am. Chem. Soc. 2014, 136, 7428–7434. 10.1021/ja5025047. 24766458

[ref131] Yonekura K.; Kato K.; Ogasawara M.; Tomita M.; Toyoshima C. Proc. Natl. Acad. Sci. U. S. A. 2015, 112, 3368 10.1073/pnas.1500724112. 25730881 PMC4372003

[ref132] Yonekura K.; Matsuoka R.; Yamashita Y.; Yamane T.; Ikeguchi M.; Kidera A.; Maki- Yonekura S. IUCrJ 2018, 5, 348–353. 10.1107/S2052252518005237. PMC5929380 29755750

[ref133] Tsirelson V. G.; Avilov A. S.; Lepeshov G. G.; Kulygin A. K.; Stahn J.; Pietsch U.; Spence J. C. H. J. Phys. Chem. B 2001, 105, 5068–5074. 10.1021/jp0015729.

[ref134] Avilov A.; Lepeshov G.; Pietsch U.; Tsirelson V. J. Phys. Chem. Solids 2001, 62, 2135–2142. 10.1016/S0022-3697(01)00170-6.

[ref135] Non-innocent ligand. Wikipedia. https://en.wikipedia.org/wiki/Non-innocent_ligand (accessed 17/07/2021).

[ref136] Engelhardt F.; Maas̈ C.; Andrada D. M.; Herbst-Irmer R.; Stalke D. Chem. Sci. 2018, 9, 3111–3121. 10.1039/C7SC05368A. 29732094 PMC5916014

[ref137] Niepötter B.; Herbst-Irmer R.; Kratzert D.; Samuel P. P.; Mondal K. C.; Roesky H. W.; Jerabek P.; Frenking G.; Stalke D. Angew. Chem., Int. Ed. 2014, 53, 2766–2770. 10.1002/anie.201308609. 24481811

[ref138] Parsons S. Acta Crystallogr., Sect. D: Biol. Crystallogr. 2003, D59, 1995–2003. 10.1107/S0907444903017657. 14573955

[ref139] Mugnaioli E.; Gorelik T. E. Acta Crystallogr., Sect. B: Struct. Sci., Cryst. Eng. Mater. 2019, B75, 550–563. 10.1107/S2052520619007339. 32830712

[ref140] Andrusenko I.; Hamilton V.; Mugnaioli E.; Lanza A.; Hall C.; Potticary J.; Hall S. R.; Gemmi M. Angew. Chem., Int. Ed. 2019, 58, 10919–10922. 10.1002/anie.201904564. 31210373

[ref141] Feyand M.; Mugnaioli E.; Vermoortele F.; Bueken B.; Dieterich J. M.; Reimer T.; Kolb U.; de Vos D.; Stock N. Angew. Chem., Int. Ed. 2012, 51, 10373–10376. 10.1002/anie.201204963. 22976879

[ref142] Gruene T.; Li T.; van Genderen E.; Pinar A. B.; van Bokhoven J. A. Chem. - Eur. J. 2018, 24, 2384–2388. 10.1002/chem.201704213. 29193398

[ref143] Kolb U.; Matveeva G. N. Z. Z. Kristallogr. - Cryst. Mater. 2003, 218, 259–268. 10.1524/zkri.218.4.259.20741.

[ref144] Pennisi E. 2018 Breakthrough of the Year. https://vis.sciencemag.org/breakthrough2018/ (accessed 20/07/2021).

